# Supplementary material for: Deep kernel learning of dynamical models from high-dimensional noisy data
Source: Sci Rep. 2022 Dec 13;12:21530. doi: 10.1038/s41598-022-25362-4 (PMC9747975; doi:10.1038/s41598-022-25362-4)
Supplement: Supplementary file 1 — Supplementary Information. [file 41598_2022_25362_MOESM1_ESM.pdf]

A Implementation Details

The proposed SVDKL framework is constructed using the GPyTorch[1] library.

A.1 SVDKL-AE Architecture

The input measurements are $84 \times 84 \times 3$ RGB images. To allow the encoder to learn the angle of the pendulum and its angular velocity, two consecutive frames are stacked together, making the input measurements of size $84 \times 84 \times 6$. The SVDKL encoder E is composed of 4 convolutional layers with 32 filters per layer. The convolutional filters are of size (3×3) and shifted across the images with stride 1 (only the first convolutional layer has stride 2 to quickly reduce the input dimensionality). Batch normalization is also used after the 2nd and 4th convolutional layers. A similar convolutional architecture is used in PlaNet[2] and Dreamer[3]. The output features of the last convolutional layer are flattened and fed to two final fully connected layers of dimensions 256 and 20, respectively, compressing the features to a 20-dimensional feature vector. Each layer has ELU activations, except the last fully-connected layer with a linear activation.

The latent variables of the feature vector are fed to independent GPs with constant mean and ARD-SE kernel, which produces a 20-dimensional latent state distribution $p(\mathbf{z}_t|\mathbf{x}_t)$. From the latent state distribution $p(\mathbf{z}_t|\mathbf{x}_t)$, we can sample the latent state vectors $\mathbf{z}_t$ using the reparametrization trick[4].

Similar to VAEs, the latent state vectors $\mathbf{z}_t$ are fed into the decoder D to learn the reconstruction distribution $p(\hat{\mathbf{x}}_t|\mathbf{z}_t)$. A popular choice for $p(\hat{\mathbf{x}}_t|\mathbf{z}_t)$ is Gaussian with unit variance[2,3,5]. The decoder D is parametrized by an NN composed of a linear fully-connected layer and 4 transpose convolutional layers with 32 filters each. The convolutional filters are of size (3×3) and shifted across the images with stride 1 (again, the last convolutional layer has stride 2). Batch normalization is used after the 2nd and 4th convolutional layers, and ELU activations are employed for all the layers except the last one. The outputs are the mean $\mu_{\hat{x}}$ and variance $\sigma^2_{\hat{x}_t}$ of $\mathcal{N}(\mu_{\hat{x}_t}, \sigma^2_{\hat{x}_t})$.

A.2 SVDKL Dynamical Model Architecture

Given a sample $\mathbf{z}_t$ from the latent state distribution $p(\mathbf{z}_t|\mathbf{x}_t)$, we predict the evolution of the dynamical system forward in time with a control input u using the SVDKL dynamical model F. The SVDKL dynamical model is composed of 3 fully-connected layers of size 512, 512, and 20, respectively, with ELU activations except the final layer with a linear activation. Analogously to the SVDKL encoder, the output features of the neural network are fed to 20 independent GPs to produce a 20-dimensional next state distribution $p(\mathbf{z}_{t+1}|\mathbf{z}_t, \mathbf{u}_t)$. Again, we sample the next latent states $\mathbf{z}_{t+1}$ using the reparametrization trick.

A.3 Pendulum Environment

The pendulum environment used for collecting the data tuples is the `Pendulum-v1` from Open-Gym[6].

A.4 KL Balancing

Similar to Dreamer-v2[5], we employ the KL balancing. The method allows for balancing how much the prior is pulled towards the posterior and vice versa, and can be easily implemented as follows:

$$\mathcal{L}_{KL} = \mathbb{E}_{\mathbf{x}_t, \mathbf{x}_{t+1} \sim \mathbf{X}, \mathbf{u}_t \sim \mathbf{U}} \Big[\alpha \mathrm{KL}\big[\mathrm{stop\_grad}(p(\mathbf{z}_{t+1}|\mathbf{x}_{t+1})) || p(\mathbf{z}_{t+1}|\mathbf{z}_t, \mathbf{u}_t)\big] + (1-\alpha)\mathrm{KL}\big[p(\mathbf{z}_{t+1}|\mathbf{x}_{t+1}) || \mathrm{stop\_grad}(p(\mathbf{z}_{t+1}|\mathbf{z}_t, \mathbf{u}_t))\big] \Big],$$

where α is a hyperparameter balancing the contribution of the two terms of the KL divergence, and stop_grad is the function stopping the propagation of the gradients during the update step of the SVDKL parameters.

A.5 Hyperparameter Summary

The hyperparameters are chosen via grid search among the values reported in Table 1. The final values used in the experiments are indicated in bold. Other parameters used in the experiments are listed in Table 2.

B Comparison with Variational Autoencoder

We compare the proposed SVDKL-based scheme with a VAE-based counterpart[4] in the low-dimensional learning of state representation and latent forward model (for the pendulum) using high-dimensional noisy measurements.

B.1 Architecture

For a fair comparison, the VAE- and SVDKL-based schemes have very similar model architectures. We use the same encoding architecture (see Section A.1), and the two models only differ in the last layer of encoder, i.e., the outputs of the VAE are the means and standard deviations of the Gaussian distributions for latent states. Similarly, the NN-based latent forward models are formulated identically (see Section A.2), expect that the means and standard deviations of the next latent states are defined as outputs in the VAE-based scheme.

Hyperparameter	Value
learning rate of NN	$[1e{-}5, 1e{-}4, 2e{-}4, \mathbf{3e-4}, 4e{-}4, 5e{-}4, 1e{-}3, 1e{-}2]$
learning rate of GP	$[1e{-}1, \mathbf{1e-2}, 1e{-}3]$
L^2 regularization coefficient	$[1e{-}1, \mathbf{1e-2}, 1e{-}3, 1e{-}4, 1e{-}5]$
α	$[0.8, \mathbf{0.9}, 1.0]$
β	$[\mathbf{1.0}]$
latent state dimension	$[5, 10, \mathbf{20}, 50]$
number of inducing points	$[\mathbf{32}, 64]$

Table 1. Hyperparameters in the proposed SVDKL-based scheme. **Bold font** indicates the actual value used for generating the results.

Other parameter	Value
image dimension	$84 \times 84 \times 3$
measurement dimension	$84 \times 84 \times 3 \times 2$
control input dimension	1
mass of the pendulum	1
length of the pendulum	1
σ_x^2	$[0.1, 0.2, 0.3, 0.4, 0.5, 0.6, 0.7, 0.8, 0.9, 1.0]$
σ_u^2	$[0.2, 0.4, 0, 5, 0.6, 0.8, 1.0, 1.5]$
σ_{dyn}^2	$[0.5, 1.0, 5.0, 10.0, 50.0, 100.0, 200.0]$

Table 2. Other parameters used in numerical experiments.

B.2 Loss Function

To train the VAE-based models, we employ a VAE loss $\mathscr{L}_E(\theta_E, \theta_D)$, a dynamical model loss $\mathscr{L}_{\mathrm{F}}(\theta_F)$, and the overall loss $\mathscr{L}_{REP}(\theta_E, \theta_F, \theta_D)$ defined as their combination, all of which take the same form as their counterparts in the SVDKL-based scheme respectively, expect that there are no longer kernel hyperparameters to be determined. Because the VAE directly learns the mean and the standard deviation of the Gaussian distribution, we do not need to perform variational inference as in the case of the SVDKL-based models.

B.3 Hyperparameters

The hyperparameters for the VAE-based model training are set to the same values as in the SVDLK-based scheme, reported in Table 3.

Hyperparameter	Value
learning rate of NN	$3e-4$
L^2 regularization coefficient	$1e-2$
α	0.9
β	1.0
latent state dimension	20

Table 3. Hyperparameters in the VAE-based scheme for comparative purposes.

B.4 Results

To empirically demonstrate the advantages in using the SVDKL-based scheme over the VAE-based models for state estimation and denoising, we show the reconstructed images under different noise levels in Figure 1. The SVDKL-based models, especially in the case of high measurement noise (e.g., $\sigma_u = 0.7$ or $\sigma_x = 1.0$), provide sharper reconstructions.

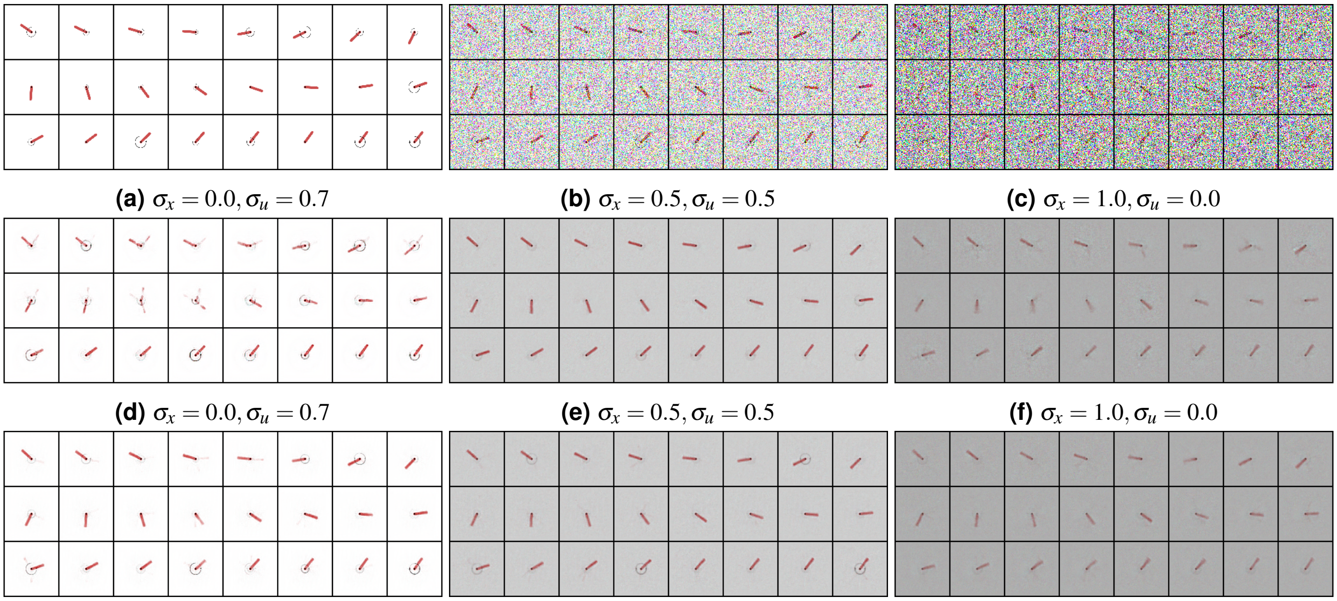

(a) $\sigma_x = 0.0, \sigma_u = 0.7$ **(b)** $\sigma_x = 0.5, \sigma_u = 0.5$ **(c)** $\sigma_x = 1.0, \sigma_u = 0.0$

(d) $\sigma_x = 0.0, \sigma_u = 0.7$ **(e)** $\sigma_x = 0.5, \sigma_u = 0.5$ **(f)** $\sigma_x = 1.0, \sigma_u = 0.0$

(g) $\sigma_x = 0.0, \sigma_u = 0.7$ **(h)** $\sigma_x = 0.5, \sigma_u = 0.5$ **(i)** $\sigma_x = 1.0, \sigma_u = 0.0$

Figure 1. Measurements (Figure 1a-1c) with different noise levels and the corresponding reconstructions through the VAE-based scheme (Figure 1d-1f) and the SVDKL-based scheme (Figure 1g-1i).

References

1. Gardner, J., Pleiss, G., Weinberger, K. Q., Bindel, D. & Wilson, A. G. Gpytorch: Blackbox matrix-matrix gaussian process inference with gpu acceleration. *Adv. neural information processing systems* **31** (2018).

2. Hafner, D. *et al.* Learning latent dynamics for planning from pixels. In *International conference on machine learning*, 2555–2565 (PMLR, 2019).

3. Hafner, D., Lillicrap, T., Ba, J. & Norouzi, M. Dream to control: Learning behaviors by latent imagination. *arXiv preprint arXiv:1912.01603* (2019).

4. Kingma, D. P. & Welling, M. Auto-encoding variational bayes. *arXiv preprint arXiv:1312.6114* (2013).

5. Hafner, D., Lillicrap, T., Norouzi, M. & Ba, J. Mastering atari with discrete world models. *arXiv preprint arXiv:2010.02193* (2020).

6. Brockman, G. *et al.* Openai gym. *arXiv preprint arXiv:1606.01540* (2016).
